# Multi-agent learning via gradient ascent activity-based credit assignment

**DOI:** 10.1038/s41598-023-42448-9

**Published:** 2023-09-14

**Authors:** Oussama Sabri, Luc Lehéricy, Alexandre Muzy

**Affiliations:** 1 CNRS, I3S, Sophia Antipolis, France; 2 CNRS, JAD, Nice, France; 3 Université Côte d’Azur, Nice, France

**Keywords:** Machine learning, Computer science

## Abstract

We consider the situation in which cooperating agents learn to achieve a common goal based solely on a global return that results from all agents’ behavior. The method proposed is based on taking into account the agents’ *activity*, which can be any additional information to help solving multi-agent decentralized learning problems. We propose a gradient ascent algorithm and assess its performance on synthetic data.

## Introduction

In multi-agent systems^1^, multiple agents aim to optimize their individual objectives, interacting with the others through these objective functions. Cooperative multi-agent systems^1,2^ aim to maximize a common objective as well.

The simplest example of single-agent reinforcement learning problem is the MAB problem^3^, in which a set of arms are presented to an agent, each associated with a reward probability distribution, unknown to the agent^4^. The agent pulls one arm at each round, and observes immediately a reward sampled from the selected arm’s probability distribution. The agent’s goal is to maximize the cumulative return over a finite number of plays. Iteratively, the agent will move towards choosing the arm with the highest return. Formally, the agent will learn an optimal policy that maximizes the expected returns.

In the Multi-Agent Multi-Armed Bandit (MAMAB) problem as defined by^5^, agents share a set of arms, and each arm provides (a possibly different) reward to each agent. This notion of MAMAB is different from ours, where each agent disposes of its own set of arms, similarly to Decentralized Partially Observable Markov Decision Processes (Dec-POMDP)^6–8^. In this paper, we focus on taking into account additional information in the learning process, in the form of an activity variable, and simplify the Dec-POMDP setting by removing the Markov evolution of the states of the agents. In other word, we consider a Dec-POMDP with a single state, with access to additional data that can be used to refine or alter the objective function.

In the context of multi-agent systems, the nature of the agents’ objective can lead to two distinct scenarios: separable and non-separable objectives. In the first case, the MAMAB can be divided into individual MAB problems, with the caveat that instead of observing their own individual reward, the agents of each MAB observe a linear combination of all individual rewards. Therefore, optimizing these individual rewards collectively maximizes the overall expected reward for the entire system. Conversely, a non-separable objective arises when the multi-agent problem defies simple decomposition into individual MAB problems. Here, the agents’ objectives are intertwined and optimizing the reward agent by agent can lead to a local optimum for the entire system. The interactions and dependencies among agents in such scenarios make it challenging for agents to promptly and individually assess their contributions to the overall objective. This problem is known as the multi-agent credit assignment problem^9^.

To solve the multi-agent credit assignment problem, we propose the Activity-based Credit Assignment (ACA)^10,11^, which has been developed to better assign credit to individual actions. In general, the activity can be any additional information that can help to modify the reward function to better fit the objective. For instance, the activity can be the cost incurred when performing the choices, be it in terms of energy, money, time, etc. It can also be an indicator of the importance of certain parts of a complex system in the execution of the action, for instance the activity of a given neuron when a subject is accomplishing a given task, or the number of calls of a function in the execution of a complex program. In ACA, the return from the environment is then computed as a function of the activity of actions and the global reward obtained at the end of an episode (a set of actions). Here, we consider the activity as a measure of the cost of the actions, in the sense that the multi-agent system wants to optimize its benefit (reward) over cost (activity) ratio. The concept of activity^12^ has been used successfully in multi-agent learning across several domains such as economics^13^, biology^14^ or cognitive sciences^15^. This work opens up a wide range of possible complex system applications for the future. More details on how the activity can be taken into account can be found in "Problem formulation" section.

We propose a policy gradient algorithm^16^ in "Policy gradient optimization" section, which is shown to converge to a zero of the gradient. Although stochastic gradient approaches converge more slowly than dedicated MAB algorithms in the framework they are designed for, gradient approaches can be shown to work in a wide variety of settings and machine learning domains^6,17,18^. This provides a general and formal way to study and ensure the convergence of the resulting algorithms, for both separable and non separable agent objectives. We evaluate the performance of our method on synthetic data in "Simulation results" section , for different forms of reward. Supplementary material contains details of the simulations (Sect. 1), the proof of the convergence theorem (Sec.2), and additional illustrations of the algorithm’s behavior (Sec.3).

ionProblem formulation

### Notations and definitions

Let $\mathbb {N}^*$ be the set of positive integers $\{1, 2, \dots \}$, and for each $n \in \mathbb {N}^*$, write [*n*] the set $\{1, \dots , n\}$.

The *multi-agent multi-armed bandit* problem, MAMAB in short, is defined as follows.

#### Definition 1

*(*MAMAB) Let $n \in \mathbb {N}^*$ be a number of multi-armed bandits (MAB for short). For each $i \in [n]$, let $k_i$ be the number of arm of agent *i* (in MAB *i*). Let $\mathcal {A}$ be the set of vectors of arms, that is $\mathcal {A}=[k_1] \times \dots \times [k_n]$.

A decision is a vector ${\textbf {a}}=(a_1,\dots ,a_n)\in \mathcal {A}$ where for all $i\in [n]$, $a_i$ is the arm pulled by agent *i*. We write $A=(A_i)_{i\in [n]}$ the random variable taking values in $\mathcal {A}$ that contains the actual decision of the agents. Its distribution is called the *policy* of the agents. Each agent picks an arm independently of the other agents, that is $\mathbb {P}(A={\textbf {a}}) = \prod _{i=1}^n \mathbb {P}(A_i = a_i)$. The marginal $\mathbb {P}(A_i = \cdot )$ is called the individual policy of agent *i*.

We consider two types of rewards.

1. *Separable reward.* When agent *i* pulls arm *a* (that is $A_i=a$), it gains a reward $R_i$ following a distribution $\mathcal {R}_{i,a}$. It also receives an additional feedback, the *activity* of the agent, which is a real-valued random variable $E_i$. Conditionally to the decision *A*, the individual reward-activity pairs $(R_1, E_1), \dots , (R_n, E_n)$ are independent, and the distributions of the reward $R_i$ and activity $E_i$ of agent *i* only depend on $A_i$, the choice made by that agent.
  To update their individual policies, the agents observe the activities $E_1, \dots , E_n$, but only have access to the *global reward* $R = \sum _{i \in [n]} R_i$, not the individual rewards. We call $E = \sum _{i \in [n]} E_i$ the total activity. In other words, *n* agents from independent MAB problems collaborate together through the shared global reward.
2. *General (non-separable) reward.* After picking decision *A*, the agents observe a reward *R*, and each agents observes its own activity $E_i$, $i=1, \dots , n$.

We call this the MAMAB problem with *n* agents, number of arms $(k_i)_{i \in [n]}$, reward distributions $(\mathcal {R}_{i,a})_{i,a}$ and activities $(E_i)_{i \in [n]}$.

**Figure 1:**
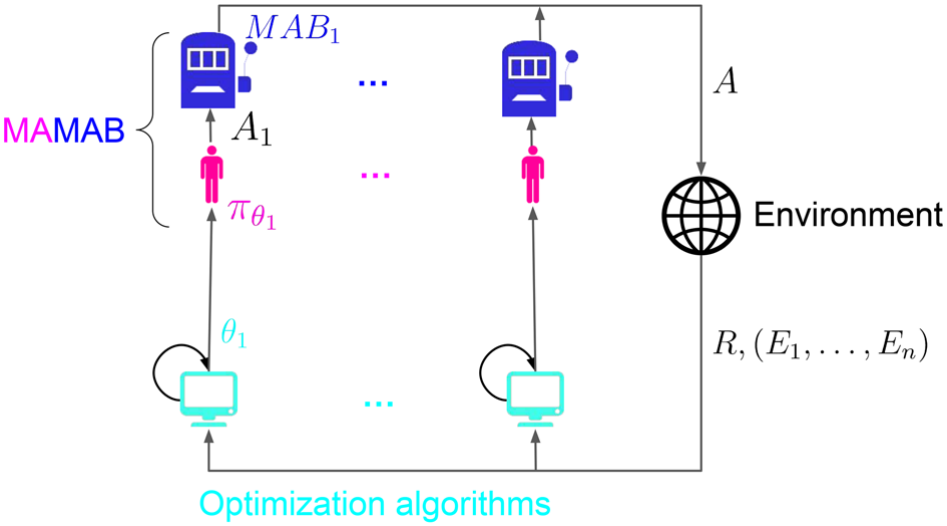
Interactions between the agents and the environment in the MAMAB, and optimization algorithm.

Figure 1 represents the interactions between the agents and the environment in the MAMAB. The optimization algorithm used to update the policy parameters is described in "GAtACA-parallel search algorithm" section.

#### Remark 1

Note the similarity of the MAMAB problem with other bandit problems:

- Adversarial Bandits with Delayed, Aggregated Anonymous Feedback (Aggregated Bandit in short), see^19^ for results on the non-adversarial version, and^20^ for results on the adversarial version when the individual rewards are observed. In Aggregated Bandits, the rewards resulting from an action can be delayed and split over several time steps. While it is possible to write a MAMAB as an Aggregated Bandit, this makes it adversarial by necessity because the delay, number of arms of the bandit, and reward distribution, depend on the time step: at time 1, the reward is delayed for $n-1$ time steps and follows the distributions of MAB 1, then at time 2, the reward is delayed for $n-2$ time steps and follows the distribution of MAB 2, and so on. This mimics the fact that the global reward is only observed at the end and makes the Aggregated Bandit formulation non-stochastic. As is, this setting also requires the numbers of arms $k_i$, $i \in [n]$, to be equal.
- Matroid Bandits, see^21^ for a definition and recent results, are a natural framework for MAMAB problems. A matroid is a finite set [*n*] and an independence system, which is a set of subsets of [*n*] satisfying nice conditions from an optimization perspective. A matroid bandit is a MAB with an additional layer of arms, the super-arms, indexed by the maximal elements of the independence system (so, these labels are sets of arms from the original MAB). Pulling a super-arm consists in pulling all the arms in the super-arm.
  The independence system $\mathcal {I}$ associated with a MAMAB is such that $X \in \mathcal {I}$ if and only if the elements of *X* are of the form (*i*, *a*) with $i \in [n]$ and $a \in [k_i]$, and for all $(i,a) \in X$ and $(j,b) \in X$, $i=j$ if and only if $a=b$. In other words, $X \in \mathcal {I}$ if it describes a subset of the *n* bandits and exactly one arm from each bandit in this subset. Unfortunately, existing works and algorithms assume that all the rewards $R_1, \dots , R_n$, are available, which is called the *semi-bandit* feedback. To our knowledge, there exists no result in the bandit setting considered here.
- A Combinatorial Bandit is a MAB with an additional layer of arms. Arms from this layer, or super-arms, are sets of arms from the original MAB, often assumed to share the same cardinality^22^. Pulling a super-arm consists in pulling all the arms it contains, and observing the sum of the rewards (bandit feedback) or the individual rewards of each arm (semi-bandit feedback). MAMABs are not a particular case of Combinatorial Bandits, as those do not respect the multi-agent structure, more precisely the fact that one and only one arm should be sampled from each MAB during each episode.

#### Definition 2

(Parameterized Policy) For each $\theta = (\theta _{1}, \dots , \theta _{n}) \in \mathbb {R}^{k_{1}} \times \dots \times \mathbb {R}^{k_{n}}$, let ${\pi _{\theta }}$ be the policy under which the random variables $(A_{1}, \dots , A_{n})$ are independent and for each $i\in [n]$ and $a \in [k_i]$,

$$\begin{aligned} {\pi _{\theta }}(A_{i} = a)=\frac{e^{\theta _{i,a}}}{\displaystyle \sum _{b\in \mathcal {A}_{i}} e^{\theta _{i,b}}}=:\textrm{softmax}(\theta _i,a), \end{aligned}$$

where for any $i\in [n]$, $\textrm{softmax}(\theta _i,\cdot )$ is the softmax function applied to $\theta _i$, which defines a probability vector on $[k_i]$. We call $\theta _i$ the *credit vector* over the actions in $[k_i]$.

Therefore, for any decision ${\textbf {a}}=(a_1,\ldots ,a_n)\in \mathcal {A}$ and any parameter $\theta$,

$$\begin{aligned} \begin{aligned} {\pi _{\theta }}(A={\textbf {a}})={\prod _{i\in [n]}}{\pi _{\theta }}(A_{i} = a_i)={\prod _{i\in [n]}}\frac{e^{\theta _{i,a_i}}}{\displaystyle \sum _{b\in \mathcal {A}_{i}} e^{\theta _{i,b}}}={\prod _{i\in [n]}}\textrm{softmax}(\theta _i,a_i ). \end{aligned} \end{aligned}$$

The goal of the agents is to maximise the expected value of a random variable *X*. We call the function *X* the *objective*. Two examples are considered in this article:

1. $X = \dfrac{R}{n}$: the objective is to maximize the global reward. In this case, if the reward *R* is separable, then the objective *X* is also separable, that is, *X* is, up to a multiplicative constant, a sum of independent random variables $R_i$ such that each depends only on one component of the decision. In this case, optimizing the global reward is equivalent to optimizing the individual rewards, which are independent optimization problems, with the difficulty coming from the fact that the individual rewards are not observed.
2. $X = \dfrac{R}{E}$: the objective is to maximize the ratio between the global reward and the total activity. For instance, if $E_i$ is the time spent while making the choice of the action $A_i$, then *X* is the reward per unit of time rather than the global reward *R*. In this case, the objective *X* is not separable.

The experiment proceeds as follows: a decision *A* is chosen according to the policy ${\pi _{\theta }}$. Then, the environment returns two feedbacks, *R* and $(E_1, \dots , E_n)$. We call one step of this procedure an *episode*, that is, sampling a decision and collecting the return. Finally, the policy parameter $\theta$ is updated based on the decision, the feedbacks, and information from past episodes. We write $A^{(e)}=(A_{i}^{(e)})_{i\in [n]}\in \mathcal {A}$ the decision sampled at episode *e*, and likewise $R_i^{(e)}$, $E^{(e)}_i$, $R^{(e)}$ and $E^{(e)}$ for all $i \in [n]$.

#### Remark 2

Conditionally to $(A^{(e)})_{e \geqslant 1}$, the feedbacks $( (E^{(e)}, R^{(e)}) )_{e \geqslant 1}$ are independent: what happens during an episode only depends on the choices made during this episode, not on what happens at other times.

### Policy gradient optimization

Write ${\mathbb {E}_{\theta }}$ the expectation under the probability distribution ${\pi _{\theta }}$.

The agents’ goal is to maximize the following objective function

1 $$\begin{aligned} \theta \longmapsto {\mathbb {E}_{\theta }}[X^{(1)}]:= {\sum _{{\textbf {a}}\in \mathcal {A}}} {\pi _{\theta }}(A^{(1)}={\textbf {a}}){\hspace{0.1cm}} \lambda _{\textbf {a}}, \end{aligned}$$

where *X* is the objective and $\lambda _{\textbf {a}}=\mathbb {E}[X^{(1)} \,\vert \, A^{(1)}={\textbf {a}}]$. Note that this conditional expectation does not depend on $\theta$: it is a function of the environment and the decision, not of the policy.

#### Theorem 1

Let $B^{(1)}$ be a random variable that is independent of $A^{(1)}$ under the parameter $\theta$, then for all $i\in [n]$ and $a\in [k_i]$,

2 $$\begin{aligned} \frac{\partial {\mathbb {E}_{\theta }}[ X^{(1)}]}{\partial \theta _{i,a}}={\mathbb {E}_{\theta }}\bigg [( \mathbbm {1}_{A_i^{(1)} = a} - \textrm{softmax}(\theta _i,a) )(X^{(1)} - B^{(1)})\bigg ]. \end{aligned}$$

This results in a gradient ascent algorithm whose convergence is ensured by the following theorem:

#### Theorem 2

Let $B^{(1)}$ be a random variable that is independent of $A^{(1)}$ under the parameter $\theta$. Assume that there exists $r>0$ such that $\vert X^{(e)}\vert \leqslant r$ and $\vert B^{(e)}\vert \leqslant r$, for all $e \geqslant 1$. Let $(\alpha _e)_{e \geqslant 1}$ be a sequence of nonnegative real numbers such that

3 $$\begin{aligned} {\sum _{e= 1}^{+\infty }} \alpha _e = +\infty {\hspace{0.5cm}}\text {and}{\hspace{0.5cm}} {\sum _{e= 1}^{+\infty }} \alpha _e^2 < +\infty . \end{aligned}$$

Update $(\theta ^{(e)})_{e \geqslant 1}$ according to the rule: for each $i\in [n]$ and $a\in [k_i]$,

4 $$\begin{aligned} \theta _{i,a}^{(e+1)} = \theta _{i,a}^{(e)} + \alpha _e (\mathbbm {1}_{A_{i}^{(e)}=a} - \textrm{softmax}(\theta _i,a)) (X^{(e)} - B^{(e)}). \end{aligned}$$

For each $$e\geqslant 1$$, $$i\in [n]$$ and $$a\in [k_i]$$, let $${\widetilde{\theta }}^{(e)}_{i,a} = \theta ^{(e)}_{i,a} - {\max _{a^\prime \in [k_i]}} \theta ^{(e)}_{i,a^\prime }$$. Then $$(\mathbb {E}_{\theta ^{(e)}}[X^{(e)}])_{e \geqslant 1}$$ converges and the limit points of $$({\widetilde{\theta }}^{(e)})_{e \geqslant 1}$$ are all in the same connected component of the set of zeroes of the gradient of $${\widetilde{\theta }} \in \Theta \longmapsto \mathbb {E}_{{\widetilde{\theta }}}[X^{(1)}]$$, where $$\Theta $$ is the (compact) set of credit vectors $${\widetilde{\theta 
}}$$ such that $${\widetilde{\theta }}_{i,a} \in [-\infty ,0]$$ and $${\max _{a^\prime \in [k_i]}} {\widetilde{\theta }}_{i,a^\prime } = 0$$ for all *i* and *a*.

The two theorems above are proved in the supplementary materials (Sect. 2).

#### Remark 3

With this algorithm, the update of the policy of agent *i*, which only depends on $\theta _i$, relies only on agent *i*’s actions and on the feedback $X^{(e)}$: both the policy and its update scheme are fully decentralized, each agent makes decisions and updates its decision process independently of the others.

### GAtACA-parallel search algorithm

Substituting one sample of the expectation in Equation (2) to estimate the gradient leads to the updating scheme in Equation (4) for some positive sequence $(\alpha _e)_{e\geqslant 1} \subset \mathbb {R}^{+}$. We choose the baseline as a discounted mean: $B^{(e)} = \dfrac{1-\gamma }{1-\gamma ^{e-1}} {\sum _{k=1}^{e-1}}\gamma ^{e-k-1}X^{(k)}$, with $B^{(1)}=0$, and $\gamma =0.99$. This algorithm and choice of baseline are similar to the REINFORCE algorithm introduced in^23^.

Algorithm 1 describes the general algorithm called GAtACA-Parallel. The parallel property refers to the fact that at each time, agents take their decision in parallel.
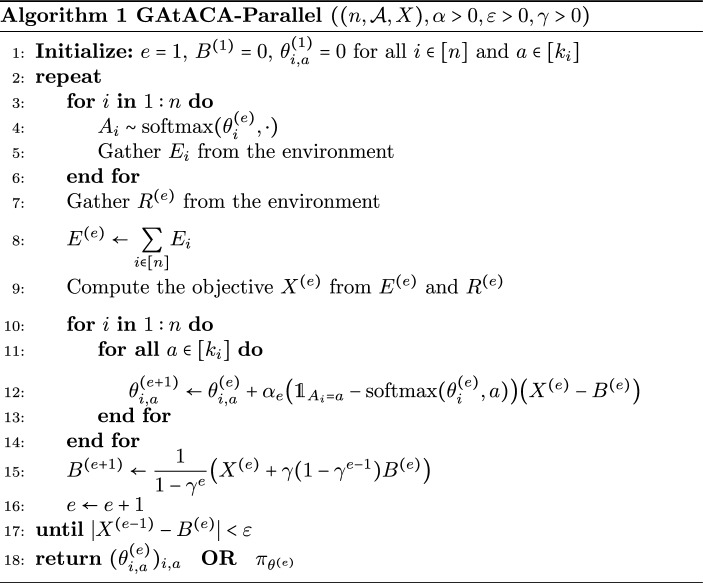


As illustrated in Fig. 1, each agent *i* uses its own optimization algorithm, GAtACA-Parallel, independently from other agents to update its policy $\pi _{\theta _i}$. This is a decentralized policy where each agent relies only on a partial knowledge of the other agents’ actions^24^ through the global reward and total activity.

## Simulation results

To guarantee the convergence, with probability one, to a zero of the gradient of the objective function, Theorem 2 states that the sequence $(\alpha _e)_{e\geqslant 1}$ must satisfy the Robbins-Monro conditions in Equation (3)^25,26^. This is a usual assumption in policy gradient algorithms. Nevertheless, how to choose this sequence in practice is a delicate issue with no universal answer. Instead, we use a small constant learning rate ($\alpha =10^{-2}$) that works well in practice. Figure 1 of supplementary materials shows learning curves for more values of $\alpha$. Despite not being covered by the above theorem, the simulations in this section show that the algorithm does approach an optimal solution.

The MAMAB parameters are as follows: there are $n=30$ agents and the number of actions of each agent $k_i$ is drawn randomly in $[\![2;7]\!]$. We consider a deterministic distribution for $R_i$ and $E_i$ conditionally to the actions, that is, for all $i\in [n]$ and $a\in [k_i]$, $R_i = \sum _{a \in [k_i]} \mu _{i,a} \mathbbm {1}_{A_i=a}$ (so that it is separable) and $E_i = \sum _{a \in [k_i]} \nu _{i,a} \mathbbm {1}_{A_i=a}$. Only a subset of $n'$ MAB contributes to the global reward: a set $\mathcal {I}\subset [n]$ of size $n'=20$ is sampled at random, and for all $i \in [n] \setminus \mathcal {I}$ and $a \in [k_i]$, $\mu _{i,a} = \nu _{i,a} = 0$. For the other agents $i \in \mathcal {I}$, the values $\mu _{i,a}$ and $\nu _{i,a}$ are drawn independently from a uniform distribution in $[-1,1]$ and [0, 1] respectively. Each experiment is run for $10^5$ episodes. Further details about the implementation are provided in supplementary materials (Sect. 1).

### Separable objective

**Figure 2:**
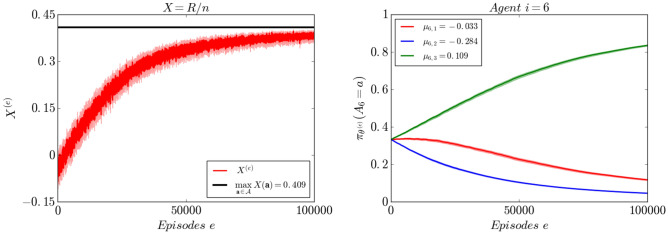
Evolution of the objective $X^{(e)}=\frac{R^{(e)}}{n}$ (left) and agent $i=6$’s policy $\pi _{\theta ^{(e)}}(A_i=\cdot )$ (right). The objective *X* is increasing and converges asymptotically to its maximum (solid line), as the agent reinforces the action with the highest return. The curves are averaged over 30 realizations, the $95\%$ confidence intervals are shown in lighter color.

Figure 2 shows the evolution of GAtACA-Parallel algorithm when the objective function is $X^{(e)} = R^{(e)}/n$.

Figure 2 (right), shows the evolution of the individual policy of agent $i=6$ (among the 30 agents). This agent is one of the agents contributing to the global reward. The probability of the action with the highest contribution to *X*, the action $a=3$, increases to 1: this agent’s policy converges toward the optimal one.

**Figure 3:**
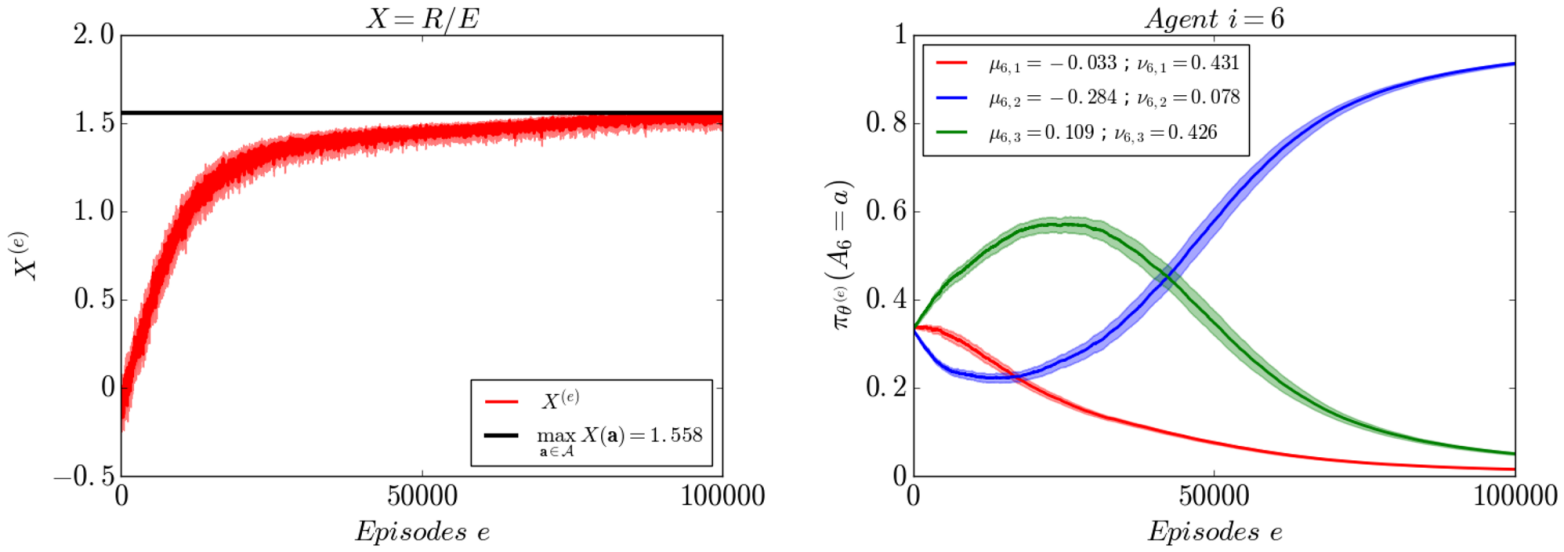
Evolution of the multi-agent objective $X=\frac{R}{E}$ (left) and agent $i=6$’s policy $\pi _{\theta ^{(e)}}(A_i=\cdot )$ (right). The objective *X* is increasing and converges asymptotically to its maximum (solid line). The curves are averaged over 30 realizations, the $95\%$ confidence intervals are shown in lighter color.

The case where an agent does not contribute to the global reward will see its policy parameters, and *a fortiori* its individual policy, remain stationary (see Fig. 3 for agent $i=30$ in supplementary materials). This is due to the cancellation of the updates on the parameters of $(\theta _{i,a})_{a \in [k_i]}$ with the same average reward through episodes, on average. When $\alpha$ is sufficiently small, the amount of the gradient added to an action parameter $\theta _{i,a}$ during the update, when this action is selected, will be substituted when *a* is not selected in the upcoming updates. More generally, if several actions from a agent are optimal, then the algorithm will assign a probability that tends to 1 to the set of optimal actions but will not favour any one of them over the others.

### Non-separable objective

We consider the same setting as in "Separable objective" section  with the objective function $X=\frac{R}{E}$. All the agents learn the optimal policy and the objective converges asymptotically toward its maximum. An interesting behavior when the objective *X* is non-separable is that the probability that an agent takes a given action is not always monotone. In fact, as the learning proceeds, each agent corrects its behavior as the other agents are learning. In Fig. 3, agent $i=6$ reinforces action $a=3$ (in green) first before it changes direction and reinforces action $a=2$ (in blue). Since the objective *X* is a ratio between the global reward *R* and the total activity *E*, the optimal trajectory is neither the maximum of *R* nor the minimum of *E* (See Fig. 2 in supplementary materials).

## Conclusion and perspectives

We proposed a general gradient-based policy approach to solve the credit assignment problem in the case of cooperative MAMABs. In this model, agents make decisions independently of the other agents’ choices, and get a reward that takes all their actions into account. The, they seek to optimize their individual policy to maximize the shared reward. In addition to the shared reward, each agent has access to a personal, additional information, its activity. We compare two cases, one where the activity is ignored and one where the agents aim to maximize the ratio of the reward and the total activities of all agents.

We proposed a gradient-based policy GAtACA-Parallel algorithm to solve this cooperative MAMAB problem for any form of multi-agent objective function *X*. Its convergence to a zero of the gradient holds for properly chosen learning rates.

A possible perspective is to study under which conditions the GAtACA-Parallel algorithm converges towards the globally optimal solution, and not merely a zero of the gradient, as well as its algorithmic computational complexity, and its rate of convergence.

Another application is to extend the use of activity for more general RL models, starting with Decentralized POMDP^6–8^.

### Supplementary Information

Supplementary Information.

## Data Availability

The datasets used and/or analysed during the current study available from the corresponding author on reasonable request.

## References

[1] Weiss G (2013). Multi-agent Systems: A Modern Approach to Distributed Artificial Intelligence.

[2] Panait L, Luke S (2005). Cooperative multi-agent learning: The state of the art. Auton. Agent. Multi-Agent Syst..

[3] Slivkins A (2019). Introduction to multi-armed bandits. Found. Trends Mach. Learn..

[4] Auer P, Cesa-Bianchi N, Fischer P (2002). Finite-time analysis of the multiarmed bandit problem. Mach. Learn..

[5] Hossain S, Micha E, Shah N (2021). Fair algorithms for multi-agent multi-armed bandits. Adv. Neural. Inf. Process. Syst..

[6] Foerster, J., Farquhar, G., Afouras, T., Nardelli, N. & Whiteson, S. Counterfactual multi-agent policy gradients. In *Proceedings of the AAAI Conference on Artificial Intelligence*, Vol. 32, Issue 1, (2018). 10.1609/aaai.v32i1.11794.

[7] Bono G, Dibangoye JS, Matignon L, Pereyron F, Simonin O, Berlingerio M, Bonchi F, Gärtner T, Hurley N, Ifrim G (2019). Cooperative multi-agent policy gradient. Machine Learning and Knowledge Discovery in Databases.

[8] Li, Y., Xie, G. & Lu, Z. Difference advantage estimation for multi-agent policy gradients. In *Proceedings of the 39th International Conference on Machine Learning. Proceedings of Machine Learning Research*, vol. 162, (eds. Chaudhuri, K., Jegelka, S., Song, L., Szepesvari, C., Niu, G., Sabato, S.) 13066–13085. PMLR, (2022). https://proceedings.mlr.press/v162/li22w.html.

[9] Chang, Y.-H., Ho, T. & Kaelbling, L. All learning is local: Multi-agent learning in global reward games. *Adv. Neural Inf. Process. Syst.* **16** (2003).

[10] Muzy A (2019). Exploiting activity for the modeling and simulation of dynamics and learning processes in hierarchical (neurocognitive) systems. Comput. Sci. Eng..

[11] Muzy A, Zeigler BP (2017). Activity-based credit assignment heuristic for simulation-based stochastic search in a hierarchical model base of systems. IEEE Syst. J..

[12] Muzy, A., Touraille, L., Vangheluwe, H., Michel, O., Traoré, M. K. & Hill, D. R. Activity regions for the specification of discrete event systems. In *Proceedings of the 2010 Spring Simulation Multiconference*, 1–7 (2010).

[13] Muzy, A., Hill, D.R. & Zeigler, B.P. Activity-based modeling and simulation (2010).

[14] Coquillard P, Muzy A, Diener F (2012). Optimal phenotypic plasticity in a stochastic environment minimises the cost/benefit ratio. Ecol. Model..

[15] James A, Reynaud-Bouret P, Mezzadri G, Sargolini F, Bethus I, Muzy A (2023). Strategy inference during learning via cognitive activity-based credit assignment models. Sci. Rep..

[16] Sutton, R. S., McAllester, D., Singh, S. & Mansour, Y. Policy gradient methods for reinforcement learning with function approximation. *Adv. Neural Inf. Process. Syst.* **12** (1999).

[17] Defazio, A., Bach, F. & Lacoste-Julien, S. Saga: A fast incremental gradient method with support for non-strongly convex composite objectives. In *Proceedings of the 27th International Conference on Neural Information Processing Systems*. Vol. 1. NIPS’14, 1646–1654. (MIT Press, Cambridge, 2014).

[18] Bottou L (1991). Stochastic gradient learning in neural networks. Proc. Neuro-Nımes.

[19] Pike-Burke, C., Agrawal, S., Szepesvari, C. & Grunewalder, S. Bandits with Delayed, Aggregated Anonymous Feedback. arXiv (2017). 10.48550/ARXIV.1709.06853. arXiv:https://arxiv.org/abs/1709.06853.

[20] Joulani, P., Gyorgy, A. & Szepesvári, C. Online learning under delayed feedback. In *International Conference on Machine Learning*, 1453–1461, PMLR (2013).

[21] Talebi, M. S. & Proutiere, A. An optimal algorithm for stochastic matroid bandit optimization. In *Proceedings of the 2016 International Conference on Autonomous Agents and Multiagent Systems*, 548–556 (2016).

[22] Combes, R., Talebi Mazraeh Shahi, M. S. & Proutiere, A. *et al.* Combinatorial bandits revisited. *Adv. Neural Inf. Process. Syst.* **28** (2015).

[23] Williams RJ (1992). Simple statistical gradient-following algorithms for connectionist reinforcement learning. Mach. Learn..

[24] Xuan, P. & Lesser, V. Multi-agent policies: From centralized ones to decentralized ones. In *Proceedings of the First International Joint Conference on Autonomous Agents and Multiagent Systems: Part 3*, 1098–1105 (2002).

[25] Robbins H, Monro S (1951). A Stochastic Approximation Method. Ann. Math. Stat..

[26] Li, X. & Orabona, F. On the Convergence of Stochastic Gradient Descent with Adaptive Stepsizes. arXiv (2018). 10.48550/ARXIV.1805.08114. arXiv:https://arxiv.org/abs/1805.08114.

[27] Bertsekas DP, Tsitsiklis JN (2000). Gradient convergence in gradient methods with errors. SIAM J. Optim..

