# Supplementary material for: Multi-agent learning via gradient ascent activity-based credit assignment
Source: Sci Rep. 2023 Sep 14;13:15256. doi: 10.1038/s41598-023-42448-9 (PMC10502029; doi:10.1038/s41598-023-42448-9)
Supplement: Supplementary file 1 — Supplementary Information. [file 41598_2023_42448_MOESM1_ESM.pdf]

Supplementary Material: Multi-agent learning via gradient ascent activity-based credit assignment

Oussama Sabri[1,3*†], Luc Lehéricy[2,3†] and Alexandre Muzy[1,3†]

[1*]CNRS, I3S. Sophia Antipolis, France.
[2]CNRS, JAD. Nice, France.
[3]Université Côte d'Azur. Nice, France.

*Corresponding author(s). E-mail(s): ou.sabri@outlook.com;
Contributing authors: luc.lehericy@univ-cotedazur.fr;
alexandre.muzy@univ-cotedazur.fr;
†These authors contributed equally to this work.

1 Simulation environment and parameters

In this section, we present simulation results of GAtACA-Parallel. All the Monte Carlo simulations were performed over $N = 30$ realizations, using the pseudo-random number generator Mersenne Twister from of library NumPy 1.11.1, with $seed = 452361$. Each realization, indexed by $r \in [R]$, at episode $e \geqslant 1$ produces $Y^{(e),[r]}$ for any variable Y. Therefore, the mean and the standard deviation of Y over the realizations is given as

$$\forall e \geqslant 1 \longmapsto \quad \overline{Y}^{(e)} = \frac{1}{N}\sum_{r=1}^{N} Y^{(e),[r]} = \text{mean}\left((Y^{(e),[r]})_{r\in[n]} \right),$$
$$\forall e \geqslant 1 \longmapsto \quad \sigma_Y^{(e)} = \text{SD}\left((Y^{(e),[r]})_{r\in[n]} \right).$$

The confidence interval (CI) at episode e are given as follow

$$\left[\overline{Y}^{(e)} - c\frac{\sigma_Y^{(e)}}{\sqrt{N}}, \overline{Y}^{(e)} + c\frac{\sigma_Y^{(e)}}{\sqrt{N}} \right],$$

where $c \approx 2.042$ is the 97.5^{th} percentile of a student's t-distribution of degree of freedom $N-1=29$.

The same seed is used for the two cases of X considered in this paper to guaranty the same environment for different learning objective.

We provide a comprehensive list of variables used throughout the article, along with their descriptions.

**Symbol**	**Description**
n	$n \in \mathbb{N}^*$, number of agents and MABs
i	$i \in [n]$, the identity of the agent
k_i	$k_i \in \mathbb{N}^*$, number of actions at the ith MAB
A	Joint decision of the agents: $A = (A_1, \dots, A_n)$
θ_i	$\theta_i \in \mathbb{R}^{k_i}$, the *credit vector* for agent i over the actions in $[k_i]$
π_θ	The action policy: $A \sim \pi_\theta$
R	The total reward ; in the separable case,$R = \sum_{i\in[n]} R_i$
E	The total activity: $E = \sum_{i\in[n]} E_i$
$\mu_{i,a}$	The reward of action a in MAB i
$\nu_{i,a}$	The activity of agent i while taking action a in MAB i

2 Proofs

Proof **[Theorem 1]**

Recall the objective function in Eq.1 with respect to θ

$$\theta \longmapsto \mathbb{E}_\theta[X^{(1)}] = \sum_{\mathbf{a}\in\mathcal{A}} \pi_\theta(A^{(1)} = \mathbf{a})\ \lambda_{\mathbf{a}} = \sum_{\mathbf{a}\in\mathcal{A}} \left(\prod_{i\in[n]} \text{softmax}(\theta_i, \mathbf{a}_i) \right) \lambda_{\mathbf{a}},$$

where $\lambda_{\mathbf{a}} = \mathbb{E}[X^{(1)} \mid A^{(1)} = \mathbf{a}]$ is a function of the environment, and is independent of the agent's policy π_θ.

Note that for all $(i,j) \in [n]^2$

$$\frac{\partial\, \text{softmax}_{\mathcal{A}_j}(\theta_j, \mathbf{a}_j)}{\partial \theta_{i,a}} = \mathbb{1}_{i=j}\text{softmax}_{\mathcal{A}_j}(\theta_j, \mathbf{a}_j)(\mathbb{1}_{\mathbf{a}_j=a} - \text{softmax}(\theta_i, a)).$$

So that

$$\frac{\partial \pi_\theta(A^{(1)} = \mathbf{a})}{\partial \theta_{i,a}} = \pi_\theta(A^{(1)} = \mathbf{a})(\mathbb{1}_{\mathbf{a}_j=a} - \text{softmax}(\theta_i, a)).$$

Thus,

$$\begin{aligned} \frac{\partial \mathbb{E}_\theta[X^{(1)}]}{\partial \theta_{i,a}} &= \sum_{\mathbf{a}\in\mathcal{A}} \pi_\theta(A^{(1)} = \mathbf{a})(\mathbb{1}_{\mathbf{a}_i=a} - \text{softmax}(\theta_i, a))\ \lambda_{\mathbf{a}}, \\ &= \mathbb{E}_\theta[(\mathbb{1}_{A_i^{(1)}=a} - \text{softmax}(\theta_i, a))X^{(1)}], \\ &= \mathbb{E}_\theta[(\mathbb{1}_{A_i^{(1)}=a} - \text{softmax}(\theta_i, a))(X^{(1)} - B^{(1)})], \end{aligned}$$

for any random variable $B^{(1)}$ that is independent of $A_i^{(1)}$ under π_θ □

About the convergence of Theorem 2

We replace θ by $\widetilde{\theta}$ to solve convergence issues: the distributions $\pi_{\theta^{(e)}}$ and $\pi_{\widetilde{\theta}^{(e)}}$ are the same when the elements of $\theta^{(e)}$ are finite, but the limit of $(\pi_{\theta^{(e)}})_{e\geqslant 1}$ may not be well-defined when there exists i, a and $a' \neq a$ such that $\theta_{i,a}^{(e)}$ and $\theta_{i,a'}^{(e)}$ tend to $+\infty$ at the same time. In contrast, for any sequence $\widetilde{\theta}^{(e)} \longrightarrow \widetilde{\theta}^{\infty} \in \Theta$, $\pi_{\widetilde{\theta}^{(e)}} \longrightarrow \pi_{\widetilde{\theta}^{\infty}}$.

An example where the gradient is zero is when $\#\{a : \widetilde{\theta}_{i,a} = -\infty\} = k_i - 1$ for all i: in this case, the ith agent always makes the same choice of the arm. Any finite change of θ does not change the distribution of the decision chosen, and hence does not change $\mathbb{E}_\theta[X^{(1)}]$.

Note that this theorem requires the step size to tend to zero, but not too fast. This is a usual assumption in gradient descent, though how to choose $(\alpha_e)_{e\geqslant 1}$ is a delicate issue in practice with no universal answer. In our algorithm and Equation 4, we chose to take α_e constant. Despite not being covered by the above theorem, the simulations show that the algorithm does converge toward the optimal solution.

Proof **[Theorem 2]**

Proposition 3 of [27] allows to prove the convergence of algorithms of the form

$$\theta^{(e+1)} = \theta^{(e)} + \alpha_e\big(s^{(e)} + w^{(e)}\big),$$

where $s^{(e)}$ is an approximation of the gradient of a target function $\theta \mapsto f(\theta)$ in $\theta^{(e)}$ and $w^{(e)}$ is a perturbation of this gradient. In our case, $f(\theta) = \mathbb{E}_\theta[X^{(1)}]$, $s^{(e)} = \nabla f(\theta^{(e)})$ and for all i, a,

$$w_{i,a}^{(e)} = \big(\mathbb{1}_{A_i^{(e)}=a} - \text{softmax}(\theta_i^{(e)}, a)\big)\big(X^{(e)} - B^{(e)}\big) - s_{i,a}^{(e)}.$$

The assumptions of Proposition 3 of [27] on $s^{(e)}$ are clearly satisfied, and the assumptions on $w^{(e)}$ are checked by Theorem 1 and by the fact that $w^{(e)}$ is bounded by $4r$. Note that the expected reward cannot tend to $-\infty$ since it is bounded, thus their proposition ensures that the gradient at $\theta^{(e)}$ converges to zero.

Let us now show that any two limit points of the sequence are in the same connected component of the set of zeroes of the gradient. Write L the set of limit points of the sequence $(\widetilde{\theta}^{(e)})_{e\geqslant 1}$. Since $\nabla f(\widetilde{\theta}^{(e)}) = \nabla f(\theta^{(e)}) \longrightarrow 0$ and ∇f is continuous (with $f(\theta) = \mathbb{E}_\theta[X^{(1)}]$), L is a subset of the set of zeroes of the gradient. Then, writing $B(x, \varepsilon)$ the open ball of radius ε centered on x and $\overline{A}$ the closure of the set A, the definition of limit points implies the equality

$$L = \bigcap_{\varepsilon>0} \bigcap_{N\geqslant 1} \overline{\bigcup_{e\geqslant N} B(\widetilde{\theta}^{(e)}, \varepsilon)}.$$

Since $|\widetilde{\theta}^{(e+1)} - \widetilde{\theta}^{(e)}| \longrightarrow 0$, for all $\varepsilon > 0$, there exists $N(\varepsilon)$ such that $\bigcup_{e\geqslant N(\varepsilon)} B(\widetilde{\theta}^{(e)}, \varepsilon)$ is path connected, that is, there exists a continuous path between any two points of this set. Hence, it is connected (since path connectedness implies connectedness), and thus, its closure is also connected (since the closure of a connected set is also connected). Note that since it is a closed set in the compact space Θ, it is also compact. Also note that for all $\varepsilon > 0$,

$$\bigcap_{N\geqslant N(\varepsilon)} \overline{\bigcup_{e\geqslant N} B(\widetilde{\theta}^{(e)}, \varepsilon)} = \bigcap_{N\geqslant 1} \overline{\bigcup_{e\geqslant N} B(\widetilde{\theta}^{(e)}, \varepsilon)}.$$

Since an intersection of nested compact connected sets is a compact connected set, and the families $\left(\overline{\bigcup_{e \geqslant N} B(\widetilde{\theta}^{(e)}, \varepsilon)}\right)_{N \geqslant 1}$ and $\left(\bigcap_{N \geqslant 1} \overline{\bigcup_{e \geqslant N} B(\widetilde{\theta}^{(e)}, \varepsilon)}\right)_{\varepsilon > 0}$ are nested, compact sets, L is also a compact connected set, which concludes the proof.

□

3 Additional figures

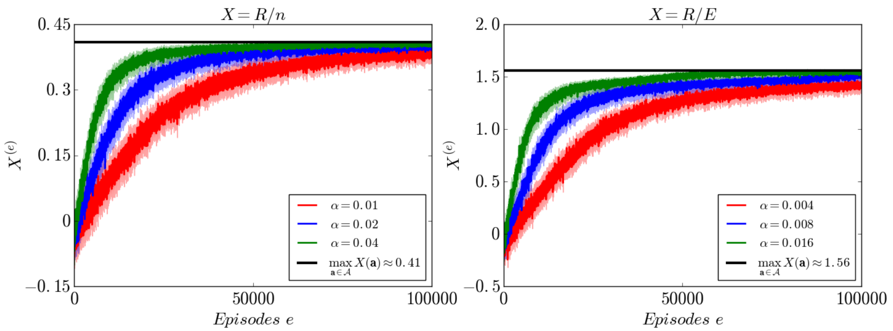

Fig. 1: Evolution of the multi-agent objective $X = \frac{R}{n}$ (left) and $X = \frac{R}{E}$ (right) for different values of α. The solid horizontal line corresponds to the maximum of X for each case.

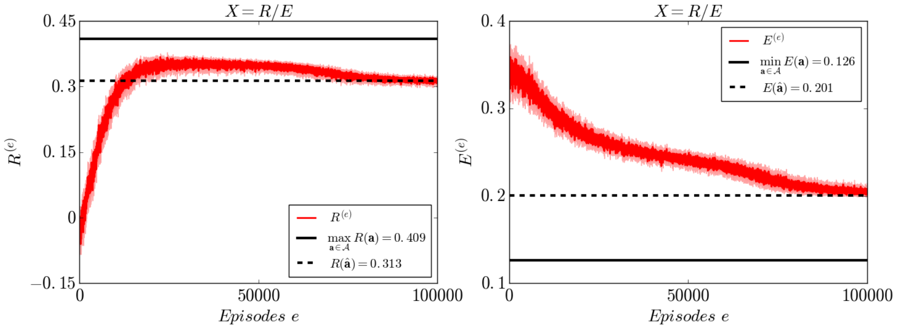

Fig. 2: Evolution of the global reward R (left) and the total activity E (right) while the objective $X = \frac{R}{E}$. The solid lines correspond to the maximum of R and minimum of the activity E. While the dashed lines correspond to the evaluation of the global return R and the total activity of the most probable trajectory $\hat{\mathbf{a}}$ under the average policy returned by the $N = 30$ GAtACA-Parallel realizations.

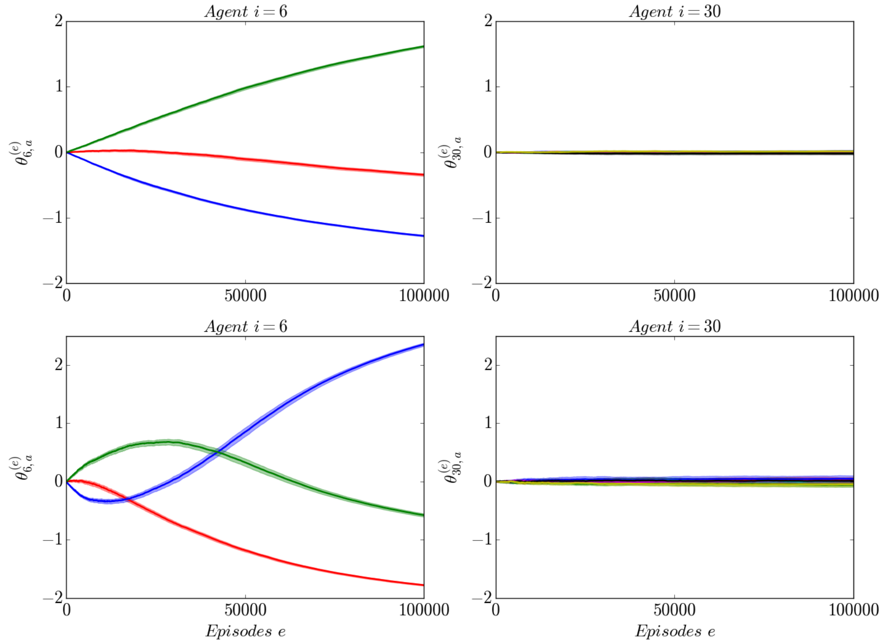

Fig. 3: Evolution of the parameter θ_i for an agent $i \in \mathcal{I}$ contributing to the global reward (agent $i = 6$) and an agent $i \in [n] \setminus \mathcal{I}$ that does not contribute (agent $i = 30$). Top: objective $X = \frac{R}{n}$. Bottom: objective $X = \frac{R}{E}$.
